# Carnitine palmitoyltransferase 1A functions to repress FoxO transcription factors to allow cell cycle progression in ovarian cancer

**DOI:** 10.18632/oncotarget.6757

**Published:** 2015-12-24

**Authors:** Huanjie Shao, Esraa M. Mohamed, Guoyan G. Xu, Michael Waters, Kai Jing, Yibao Ma, Yan Zhang, Sarah Spiegel, Michael O. Idowu, Xianjun Fang

**Affiliations:** ^1^ Departments of Biochemistry and Molecular Biology, Virginia Commonwealth University, Richmond, VA 23298, USA; ^2^ Department of Medicinal Chemistry, Virginia Commonwealth University, Richmond, VA 23298, USA; ^3^ Department of Pathology, Virginia Commonwealth University, Richmond, VA 23298, USA; ^4^ Institute of Biological Sciences, Shaanxi Normal University, Xi'an, China

**Keywords:** fatty acid β-oxidation, ovarian cancer, CPT1A, FoxO, p21

## Abstract

Cancer cells rely on hyperactive *de novo* lipid synthesis for maintaining malignancy. Recent studies suggest involvement in cancer of fatty acid oxidation, a process functionally opposite to lipogenesis. A mechanistic link from lipid catabolism to oncogenic processes is yet to be established. Carnitine palmitoyltransferase 1 (CPT1) is a rate-limiting enzyme of fatty acid β-oxidation (FAO) that catalyzes the transfer of long-chain acyl group of the acyl-CoA ester to carnitine, thereby shuttling fatty acids into the mitochondrial matrix for β-oxidation. In the present study, we demonstrated that CPT1A was highly expressed in most ovarian cancer cell lines and primary ovarian serous carcinomas. Overexpression of CPT1A correlated with a poor overall survival of ovarian cancer patients. Inactivation of CPT1A decreased cellular ATP levels and induced cell cycle arrest at G0/G1, suggesting that ovarian cancer cells depend on or are addicted to CPT1A-mediated FAO for cell cycle progression. CPT1A deficiency also suppressed anchorage-independent growth and formation of xenografts from ovarian cancer cell lines. The cyclin-dependent kinase inhibitor p21^WAF1^ (p21) was identified as most consistently and robustly induced cell cycle regulator upon inactivation of CPT1A. Furthermore, p21 was transcriptionally upregulated by the FoxO transcription factors, which were in turn phosphorylated and activated by AMP-activated protein kinase and the mitogen-activated protein kinases JNK and p38. Our results established the oncogenic relevance of CPT1A and a mechanistic link from lipid catabolism to cell cycle regulation, suggesting that CPT1A could be a prognostic biomarker and rational target for therapeutic intervention of cancer.

## INTRODUCTION

One of the most fundamental changes in cancer is the development of a lipogenic phenotype, mediated by increased expression or activity of key lipogenic enzymes primarily fatty acid synthase (FAS) and acetyl-CoA carboxylase (ACC) [1, 2]. Cancer cells rely on hyperactive lipogenesis for cell membrane biogenesis, growth and survival [1, 2]. In contrast, the role of lipid catabolism via fatty acid β-oxidation (FAO) in cancer has not been well defined. Although FAO is the major source of ATP production in normal cells, most previous studies of cancer bioenergetics have focused on abnormal glucose metabolism, namely the Warburg effect, whereby cancer cells up-regulate glycolysis to provide quick energy and biosynthetic intermediates [3, 4].

Recent evidence suggests that fatty acids from neighboring adipose tissues, lipoproteins, membrane phospholipids, and intracellular storage fat have potential to fuel cancer cells [5–9]. However, unlike glycolytic and lipogenic pathways where specific metabolic enzymes such as hexokinase 2 and FAS are known to be deregulated by oncogene (s) or by inactivation of tumor suppressors [10, 11], there is limited evidence for cancer-associated abnormal expression or activity of the enzymes directly involved in the FAO pathway. Carnitine palmitoyltransferase 1 (CPT1) is a rate-limiting enzyme of FAO that catalyzes the transfer of long-chain acyl group of the acyl-CoA ester to carnitine, thereby shuttling long-chain fatty acids into the mitochondrial matrix [12]. There are three members of the CPT1 family, CPT1A (liver form), CPT1B (muscle form) and CPT1C (brain form) encoded by three paralogous genes [12]. It has been recently reported that CPT1C is upregulated in lung cancer and is induced by metabolic stress such as hypoxia and glucose deprivation in a p53-dependent manner, potentially contributing to cytoprotection in hypoxic tumors [13, 14]. However, the fact that p53 is frequently inactivated in malignancies does not support a general role for the p53-dependent induction of CPT1C in promotion of oncogenesis. Furthermore, the brain-specific CPT1C is enzymatically deficient [12, 15, 16] or only weakly active on membranes of endoplasmic reticulum or microsomes [17]. Some biochemical studies suggest that CPT1C displays a high binding affinity with malonyl-CoA, a physiological inhibitor of CPT1 enzymes [15, 16]. Thus CPT1C could be involved in sequestering malonyl-CoA to reduce its availability to the enzymatically active CPT1A. However, a physiological role for CPT1C in fatty acid metabolism remains controversial [18, 19].

In the present study, we examined expression and biological significance of CPT1A, the most widely distributed and enzymatically active form of the CPT1 family, in human ovarian cancer that replicates and spreads within the fat-rich abdomen. Our results indicated that CPT1A is highly expressed in most ovarian cancer cell lines and ovarian serous carcinomas. Overexpression of CPT1A is associated with poor prognosis of ovarian cancer patients. Inactivation of CPT1A in ovarian cancer cell lines by pharmacological inhibition or by lentivirally-mediated knockdown led to growth arrest at G0/G1 and inhibition of tumorigenicity in SCID mice, suggesting that CPT1A-dependent FAO plays an essential role in cell cycle progression of ovarian cancer cells *in vitro* and *in vivo*. To elucidate the molecular mechanism underlying these cellular outcomes of CPT1A inhibition, we performed a PCR-based cell cycle assay and identified p21^WAF1^ (p21) as the most consistently and dramatically upregulated cell cycle regulator upon CPT1A inactivation. We further demonstrated that CPT1A inhibition induced time-dependent phosphorylation and activation of FoxO transcription factors by AMPK, JNK and p38. These results revealed a mechanistic link from CPT1A and CPT1A-dependent FAO to cell cycle regulation in ovarian cancer.

## RESULTS

### CPT1A is highly expressed and contributes to poor prognosis in ovarian cancer

**Figure 1:**
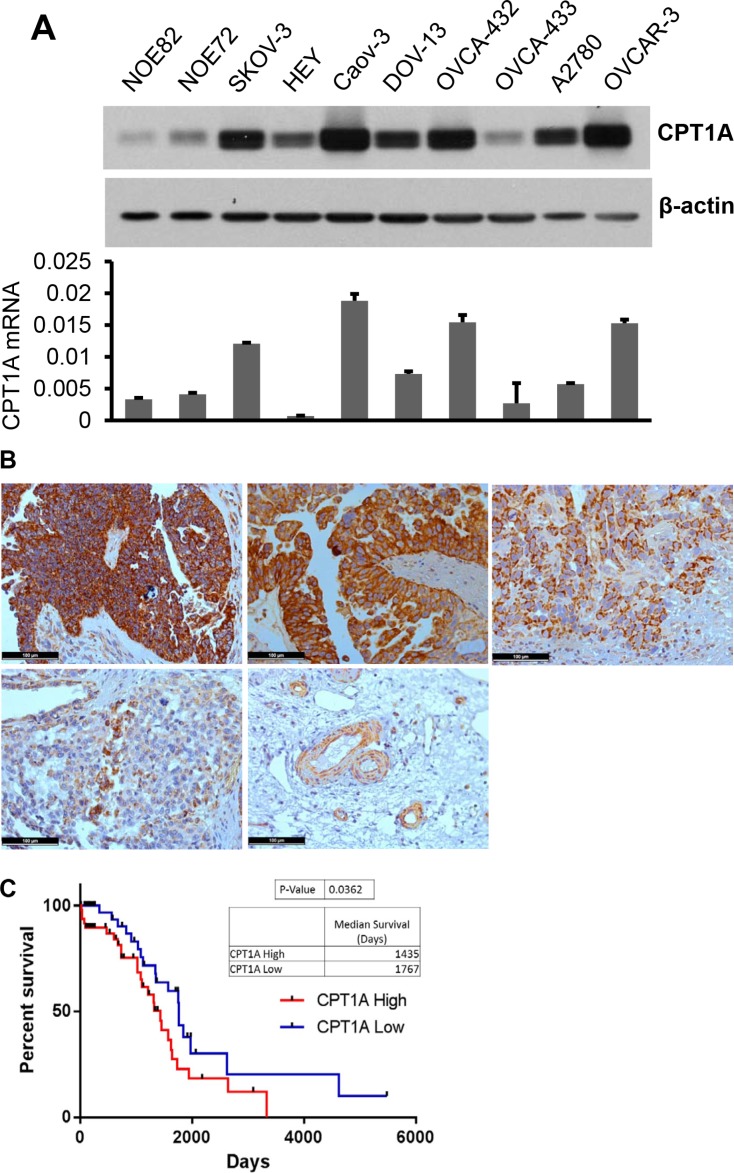
CPT1A is highly expressed in ovarian cancer and its expression correlates with poor survival of patients (**A**) Expression of CPT1A protein (***upper***) and mRNA (***lower***) in ovarian cancer cell lines was analyzed by immunoblotting and qPCR, respectively. CPT1A mRNA levels in these cells were normalized to GAPDH. (**B**) CPT1A in primary human ovarian serous carcinomas was analyzed by IHC. Shown were four patient specimens, representatives of very strong (score ≥ 12, ***upper left***), strong (score ≥ 9, ***upper middle***), modest (score ≥ 6, ***upper right***), weak (score 1–4, ***lower left***) and negative staining of non-cancerous tissues except for blood vessels (***lower right***). In (**C**) RNAseq data was downloaded from the Tumor Cancer Genome Atlas ovarian serious carcinoma dataset. Expression values were standardized via a Z-transformation by the cBioPortal for Cancer Genomics. Patients whose CPT1A expression levels were one normalized standard deviation above and below the mean level were grouped as CPTA1 High and CPTA1 Low, respectively. Kaplan Meier survival and Gehan-Breslow-Wilcoxon test of two groups were performed using GraphPad PRISM software.

To investigate the role of CPT1 and CPT1-dependent FAO in ovarian cancer, we examined expression of CPT1A in multiple ovarian cancer cell lines. Immunoblotting analysis indicated that most ovarian cancer cell lines expressed high levels of CPT1A (Figure 1A). In contrast, expression of CPT1B protein was absent in ovarian cancer cell lines (Supplementary Figure S1). We next performed qPCR analysis of CPT1A mRNA. As shown in Figure 1A, the cell lines expressing most abundant CPT1A protein such as SKOV-3, Caov-3, OVCA-432 and OVCAR-3 also showed highest levels of CPT1A mRNA, suggesting that a transcriptional mechanism mediates CPT1A overexpression in ovarian cancer cells.

We analyzed CPT1A protein expression in 18 high-grade ovarian serous carcinomas (Stage II–IV), the most common type of epithelial ovarian cancer, by immunohistochemical (IHC) staining. As shown in Figure 1B, more than half of patients showed very strong (6/18 with score ≥ 12) or strong (4/18 with score ≥ 9) staining of CPT1A in malignant epithelial cells compared to the surrounding normal tissues. Four were modestly stained (score ≥ 6) and four were weakly positive for CPT1A (score of 1–4) (Figure 1B). The results indicated that CPT1A protein is present abundantly in a high proportion of ovarian serous carcinomas.

To understand the clinical significance of CPT1A overexpression, we analyzed RNAseq data downloaded from the Tumor Cancer Genome Atlas ovarian serious carcinoma dataset (http://tcga-data.nci.nih.gov/tcga/tcgaCancerDetails.jsp?diseaseType=OV&diseaseName=Ovarian%20serous%20cystadenocarcinoma). Patients whose expression levels of CPT1A were one normalized standard deviation above the mean of CPT1A were grouped as CPT1A High. Conversely, those with one normalized standard deviation below the mean were grouped as CPT1A Low. Kaplan Meier survival and the Gehan-Breslow-Wilcoxon test of the two groups using the GraphPad PRISM software indicated that CPT1A High patients had a statistically significant shorter overall survival (medium 1435 days) than that of CPT1A Low patients (medium 1767 days) (*p =* 0.0362) (Figure 1C), suggesting that CPT1A overexpression is associated with poorer prognosis.

### Inactivation of CPT1A inhibits cell growth and ATP production

Previous studies of CPT1A in cancer have been restricted to pharmacological inhibitors such as etomoxir or its combination with inhibitors co-targeting other metabolic, oncogenic or survival cascades [20–22]. To elucidate biological functions of CPT1A in cancer cells in a more specific manner, we used lentivirus-mediated shRNA to knockdown its expression in SKOV-3, Caov-3, OVCA-432 and OVCAR-3 that expressed highest levels of endogenous CPT1A (Figure 1A). As demonstrated in Supplementary Figure S2, shRNA downregulation of CPT1A efficiently inhibited the rate of FAO, similar to the treatment of these cells with etomoxir.

**Figure 2:**
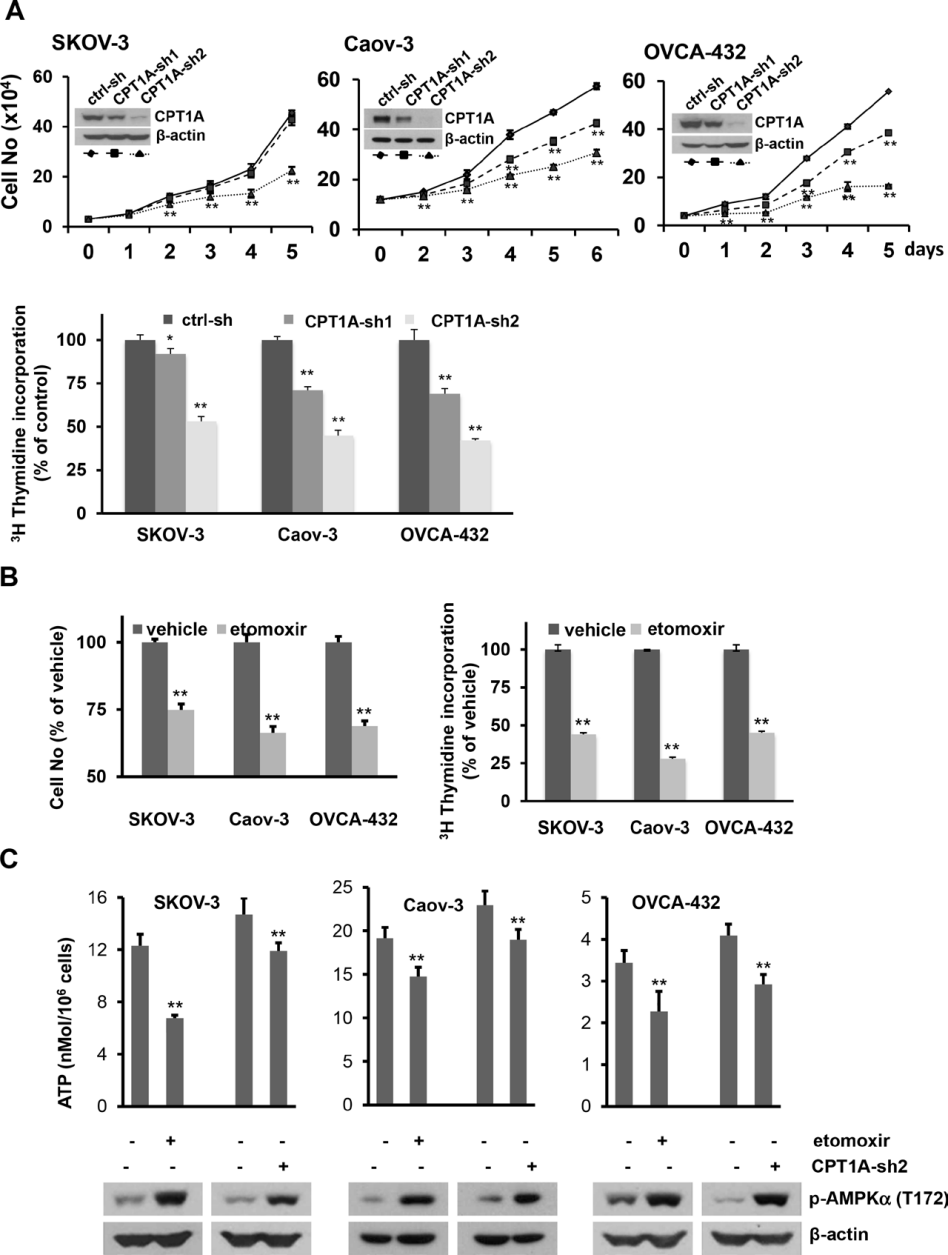
Inactivation of CPT1A decreases cellular ATP levels and cell growth (**A**) Growth curves of CPT1A-sh1, CPT1A-sh2 knockdown cells and ctrl-sh cells plated in 12-well plates were constructed from daily quantification of cell numbers with a Z1 Coulter counter (***upper***). The effect of CPT1A shRNA knockdown on cellular DNA synthesis was analyzed by [^3^H] thymidine incorporation assay as detailed in Materials and Methods. The results were normalized on cell numbers and presented as relative to ctrl-sh cells (defined as 100%). (**B**) The effects of etomoxir (0.3 mM, 24 hours) on cell numbers and [^3^H] thymidine incorporation were conducted as in (A). Results of etomoxir-treated cells were presented as % relative to values of vehicle-treated control cells (defined as 100%). **(C)** Cellular ATP levels in CPT1A knockdown cells (CPT1A-sh2), control cells (ctrl-sh), and parental cell lines treated for 24 hours with or without etomoxir (0.3 mM) were measured with an ATP bioluminescence assay kit. Subsequent activation of AMPK in these cells was assessed by immunoblotting analysis of activation-associated phosphorylation of AMPKa at T172. For this and all following figures, data are mean *+* SD of triplicates, representative of three independent experiments.

There were little or slight increases (< 4%) in apoptotic cells in association with inactivation of CPT1A (Supplementary Figure S3). A significant cellular effect we observed was inhibition of cell proliferation and cellular DNA synthesis by CPT1A inactivation (Figure 2A). The inhibition of cell proliferation correlated with knockdown efficiency of CPT1A shRNAs. CPT1A-sh2 that essentially eliminated CPT1A expression more dramatically suppressed these cells than CPT1A-sh1 that only partially downregulated CPT1A (Figure 2A). Similar to shRNA knockdown, the CPT1A inhibitor etomoxir also suppressed cellular DNA synthesis and cell proliferation (Figure 2B). Furthermore, inhibition of CPT1A and the subsequent FAO by either CPT1A shRNA or etomoxir caused prominent decreases in cellular ATP levels (Figure 2C), indicating that FAO contributes significantly to ATP production. Consistent with the drop in cellular ATP, AMP-activated protein kinase (AMPK), an energy sensor and regulator of cellular metabolism [23], was activated in CPT1A-inactivated cells as reflected by increased phosphorylation of T172 within the activation domain of the AMPK α subunit AMPKα (Figure 2C).

### CPT1A inactivation induces Go/G1 cell cycle arrest and upregulation of p21

Within a week after infection of SKOV-3, OVCA-432 and OVCAR-3 with CPT1A shRNA lentivirus, subpopulations of cells showed morphological appearances of senescence (Supplementary Figure S4). We performed staining for β-galactosidase (β-gal) activity, a biomarker of cellular senescence [24]. These early emerging, morphologically distinct cells were stained positive for β-gal. Similarly, treatment with etomoxir for 3 days also increased numbers of β-gal-positive and morphologically flattened cells (Supplementary Figure S4). However, β-gal-positive cells were not detectable in Caov-3 following CPT1A-shRNA knockdown or treatment with etomoxir (data not shown). In addition, the senescent cells became less obvious after initial passaging of CPT1A knockdown cells, suggesting that they were negatively selected and gradually eliminated from culture. As shown in Figure 2A, CPT1A knockdown cells remained to be growth inhibited although they no longer showed evident cellular senescence. Similarly, treatment with etomoxir for only 24 hours was sufficient to inhibit cell proliferation in the absence of senescence. These results suggest that replicative senescence is not the primary mechanism conferring the general growth inhibition seen in CPT1A-inactivated cells.

**Figure 3:**
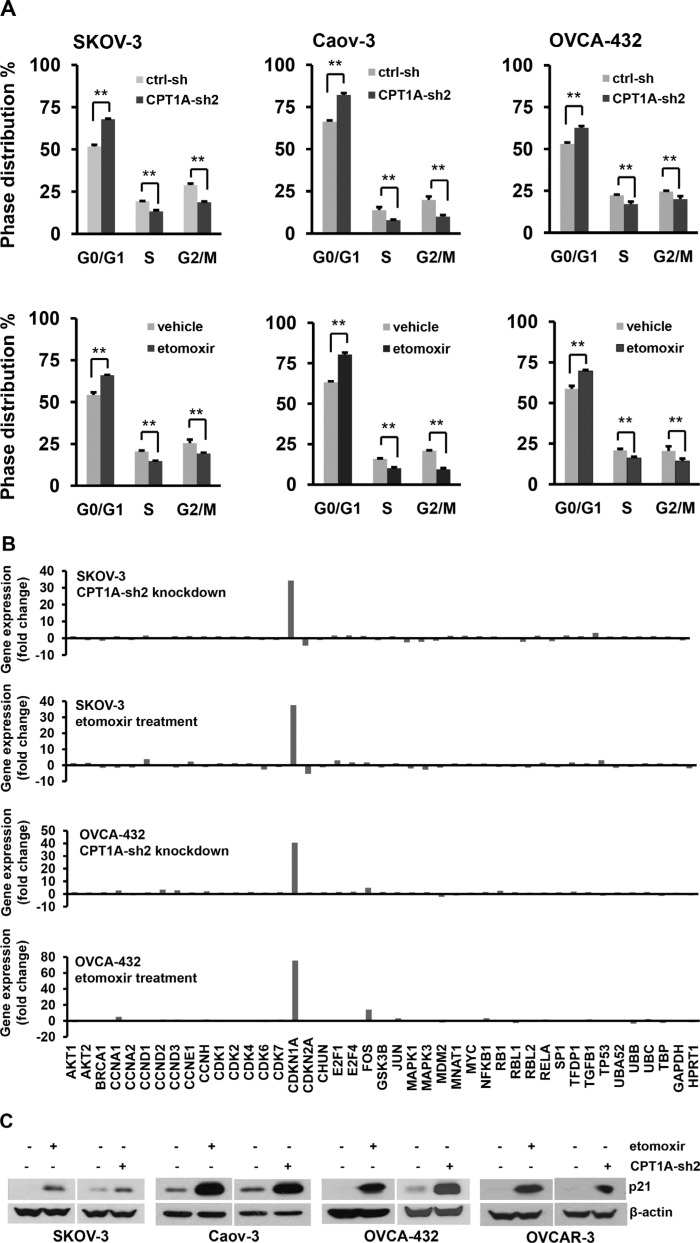
CPT1A inactivation cuases cell cycle arrest at G0/G1 and upregulation of p21 (**A**) CPT1A-sh2-tranduced cells and control (ctrl-sh) cells (***upper***), or parental cell lines treated for 24 hours with or without etomoxir (0.3 mM) (***lower***) were stained with propidium iodide and analyzed by flow cytometry. (**B**) Total cellular RNA was extracted from SKOV-3 and OVCA-432 cells and analyzed for expression of cell cycle-related genes using the Cell Cycle PrimePCR Assay as described in Methods. The relative mRNA levels of 43 genes included in the assay were normalized to that of GAPDH and presented as fold changes over the respective controls. (**C**) Induction of p21 protein by CPT1A knockdown or etomoxir was confirmed by immunoblotting.

We next conducted cell cycle analysis using flow cytometry. As shown in Figure 3A, shRNA knockdown of CPT1A or treatment with etomoxir for 24 hours induced significant increases in G0/G1 population with concomitant decreases in S and G2/M phases in all ovarian cancer cell lines examined. Therefore a major consequence of CPT1A inactivation was cell cycle arrest at G0/G1.

To identify cell cycle regulators targeted by CPT1A inhibition, we carried out a pre-designed Cell Cycle PrimePCR Assay (BioRad) in SKOV-3 and OVCA-432 cells. The assay contained 43 cell cycle-related genes and controls for normalization. Changes in gene expression were compared between CPT1A-sh2 knockdown cells and ctrl-sh cells or between etomoxir- and vehicle-treated cells. Interestingly, the cyclin-dependent kinase inhibitor p21 (CDKN1A in Figure 3B) was most drastically and consistently induced by inactivation of CPT1A in both cell lines. The expression pattern of the cell cycle-related genes in CPT1A-silenced cells was strikingly reproduced by treatment with etomoxir, suggesting that the CPT1A inhibitor targeted CPT1A specifically to recapitulate CPT1A knockdown-induced gene expression signature. In addition to p21, there were other genes that showed minor, cell line-specific up- or down-regulation. For example, p16^INK4A^ (CDKN2A) was modestly decreased by CPT1A inactivation in SKOV-3 but not in OVCA-432. Similarly, Fos was modestly induced by inactivation of CPT1A in OVCA-432 but not in SKOV-3 (Figure 3B).

We performed immunoblotting to confirm the predominant increase in p21 expression in association with CPT1A inactivation. As shown in Figure 3C, p21 was strongly induced by CPT1A-sh knockdown or etomoxir across ovarian cancer cell lines examined.

### CPT1A knockdown decreases tumorigenicity of ovarian cancer cells in SCID mice

**Figure 4:**
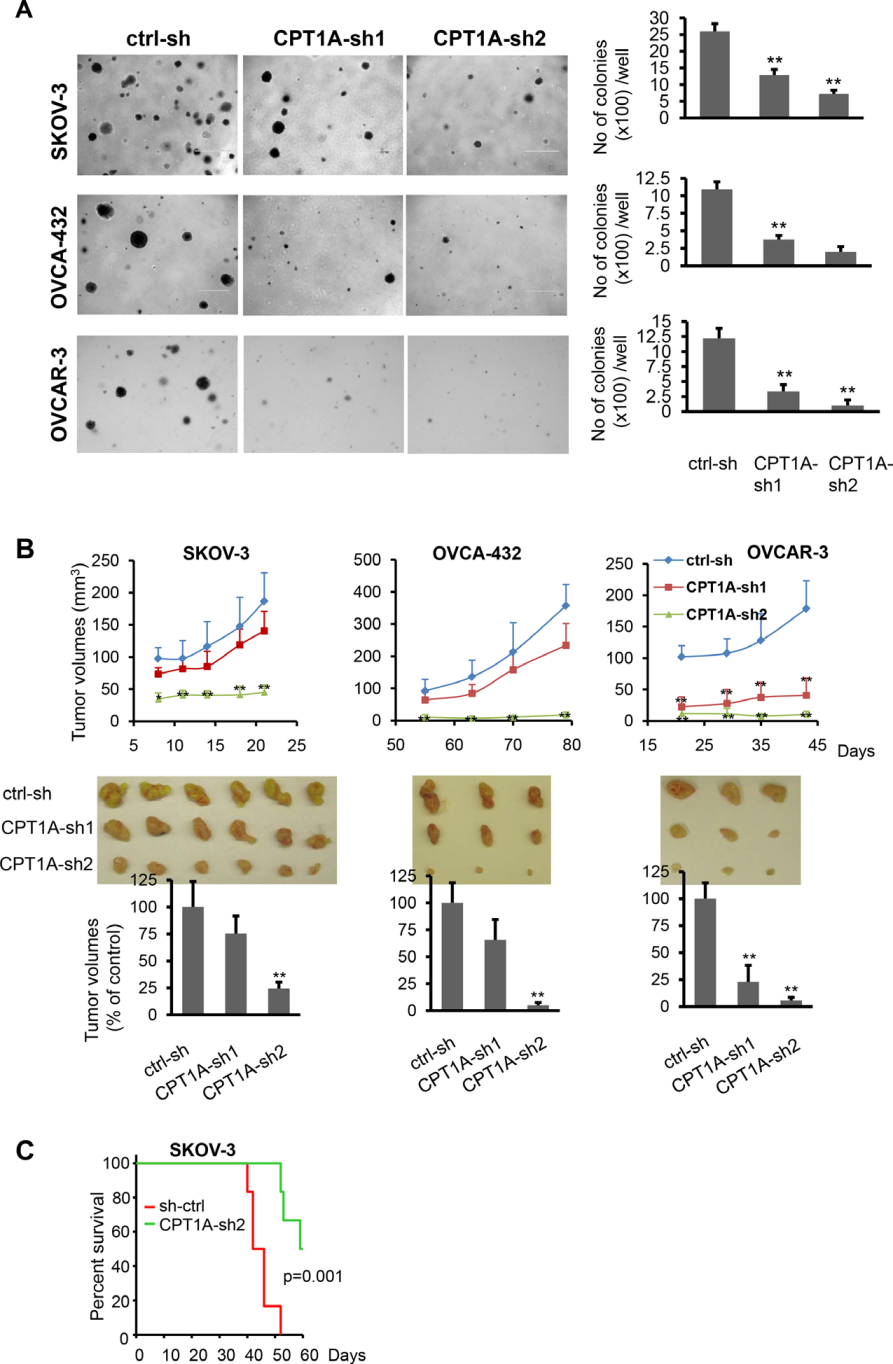
CPT1A knockdown decreases anchorage-independent growth and *in vivo* aggressiveness of ovarian cancer cells (**A**) Colony formation assay in soft agar was performed in CPT1A knockdown and control SKOV-3, OVCA-432 and OVCAR-3 cells. Microphotographs covering representative areas of each treatment were shown (***left***). Numbers of colonies (> 0.2 mm in diameter) in each well of 6-well plates were quantified (***right)***. (**B**) CPT1A knockdown and control SKOV-3, OVCA-432 and OVCAR-3 cells were injected s.c. on the right flank of SCID mice. Growth curves were constructed from tumor volumes (mean ± S.D.) measured at indicated times (days) post cell injection (***upper***). Tumors of each group were removed and photographed after sacrifice of animals (***middle***). Shown in ***lower*** panel were tumor volumes at endpoints with volumes of those grown from ctrl-sh cells defined as 100%. Statistical significances of differences between ctrl-sh xenografts and CPT1A-sh1 or CPT1A-sh2 xenografts were determined with the Student's *t*-test. (**C**) CPT1A-sh2 knockdown and control SKOV-3 cells were i.p. injected into SCID mice (6/group). Kaplan Meier survival and the LogRank test of two groups were performed using SigmaPlot 13.0.

CPT1A knockdown inhibited proliferation of ovarian cancer cells not only in monolayer culture (Figure 2A) but also in soft agar (Figure 4A). We focused on SKOV-3, OVCA-432, and OVCAR-3 cells based on their reported abilities to grow in anchorage-independent conditions and to form xenografts in mice [25, 26]. The numbers of colonies formed in soft agar were significantly reduced in CPT1A shRNA knockdown cells. We next examined the effect of shRNA knockdown on tumorigenicity of these cells in SCID mice. CPT1A-sh2 knockdown also strongly inhibited initiation and enlargement of subcutaneous xenografts in SCID mice as reflected by their growth curves (Figure 4B). Consistent with this, mice intraperitoneally (i.p.) injected with CPT1A-sh2 knockdown SKOV-3 cells survived significantly longer than those injected with control cells as indicated by Kaplan Meier survival analysis (Figure 4C).

### CPT1A inhibition induces p21 expression through a FoxO-dependent mechanism

**Figure 5:**
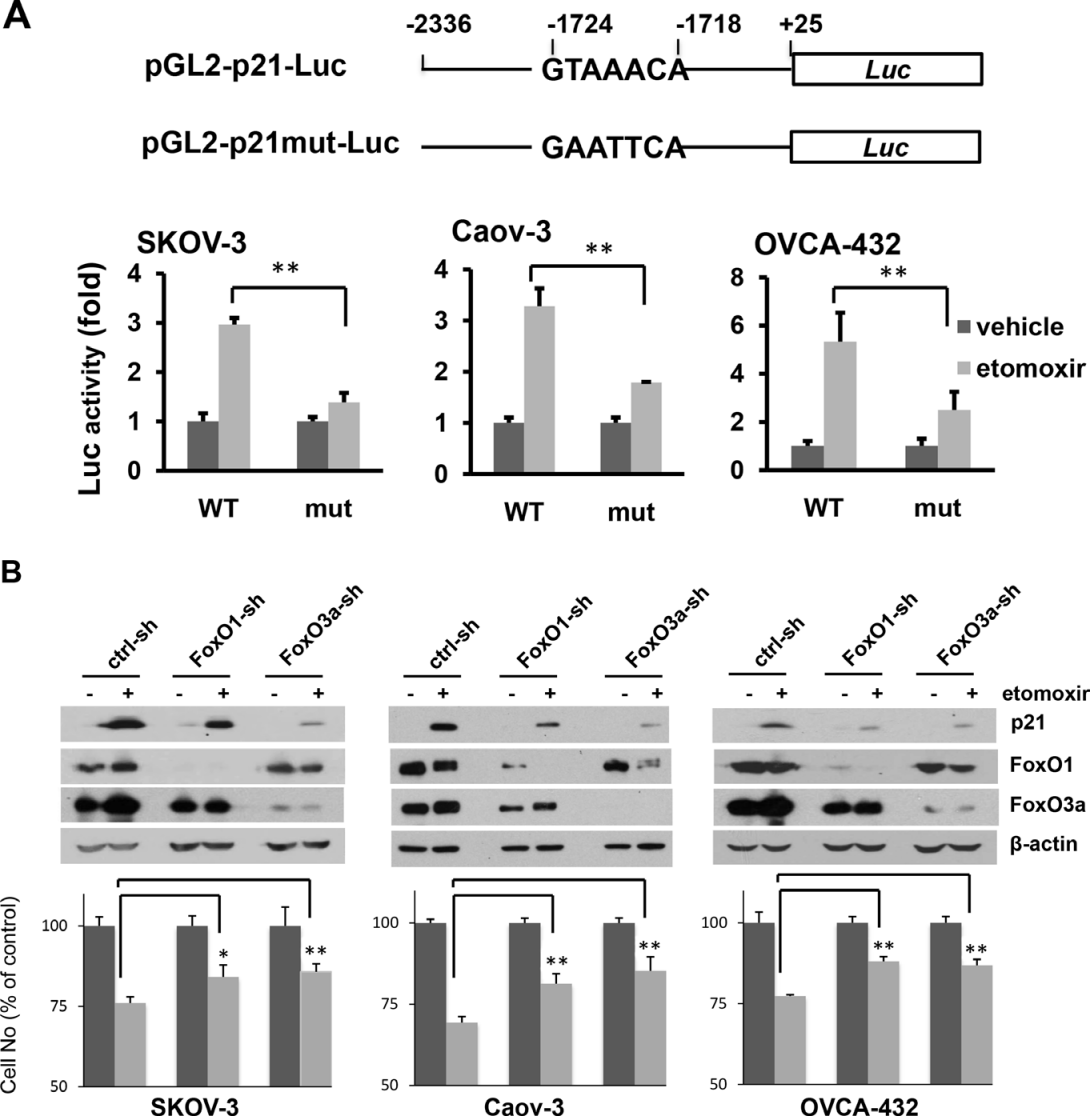
Inhibition of CPT1A induces p21 expression through a FoxO-dependent mechanism (**A**) The p21 gene promoter (−2336 to +25) was cloned into pGL2-Basic-Luc to construct the pGL2-p21-Luc reporter (WT). The FoxO mutant form pGL2-p21mut-Luc was made by mutation of the FoxO site (−1724 to −1728) (***upper***). Ovarian cancer cell lines were transfected with the indicated plasmids and incubated with or without etomoxir (0.3 mM) for 24 hours before luciferase activities were determined. The results were presented as fold changes relative to the values of the vehicle control cells (defined as 1) ***(lower***). (**B**) The effects of shRNA knockdown of FoxO1-sh or FoxO3a-sh on induction of p21 (***upper***) and inhibition of cell proliferation (***lower***) by etomoxir (0.3 mM, 24 hours) were examined. The results of cell numbers were presented as values relative to control cells untreated with etomoxir (defined as 100%).

Transcription of p21 is tightly regulated by a variety of transcription factors, most notably p53 [27]. However, many ovarian cancer cell lines including SKOV-3, Caov-3 and OVCAR-3 used in this study were known to lack p53 or carry mutant p53 [28], suggesting a p53-independent induction of p21 to respond to inactivation of CPT1A. To explore the underlying mechanism, we examined the effect of CPT1A inhibition on the p21 gene promoter using a luciferase reporter assay. Treatment with etomoxir caused multi-fold increases in luciferase activity driven from a 2.3-kb fragment of the human p21 gene promoter [27, 29] (Figure 5A). There are a number of cis elements within the promoter sequence, including a well-defined FoxO binding site GTAAACA located at −1724/−1718 [30]. Recent studies have shown involvement of FoxO transcription factors in regulating p21 expression in response to metabolic and endocrine cues [30–32].

When the FoxO-binding site was mutated to GAATTCA, activation of the p21 gene promoter by etomoxir was blocked (Figure 5A), suggesting involvement of FoxO transcription factors in upregulation of p21 in CPT1A-inactivated cells. FoxO1 and FoxO3a were ubiquitously expressed in ovarian cancer cell lines (Figure 5B). In further support of FoxO-dependent mechanism to induce p21, knockdown of FoxO1 or FoxO3a attenuated upregulation of p21 and inhibition of proliferation by etomoxir (Figure 5B).

### Inhibition of CPT1A stimulates phosphorylation and activation of FoxO transcription factors

FoxO proteins are phosphorylated by a multitude of protein kinases including AKT, AMPK, ERK, JNK and p38 kinases [33]. FoxO phosphorylated by AKT binds to 14–3–3 proteins, resulting in cytosolic localization and inactivation of FoxO [34]. Thus FoxO could be activated through inhibition of AKT. However, we did not detect any decreases in phosphorylated AKT or AKT-mediated phosphorylation of FoxO (T24/T32) in CPT1A-inactivated cells (data not shown).

**Figure 6:**
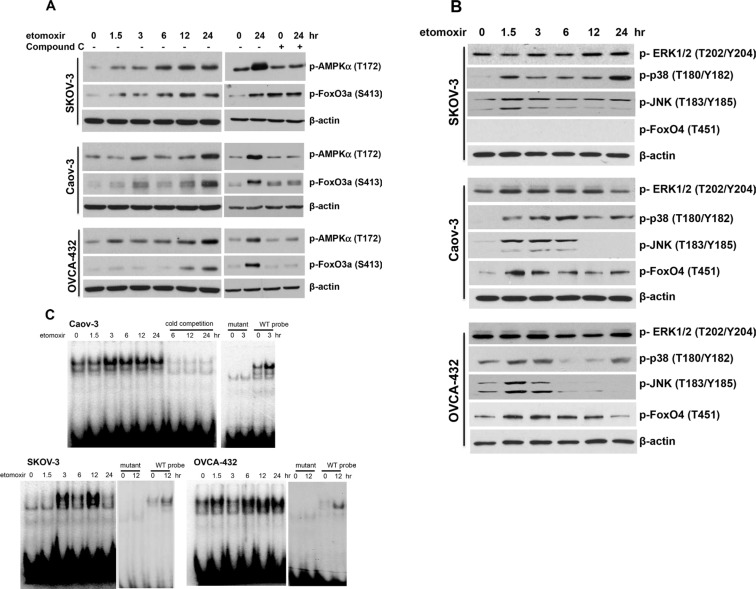
Inhibition of CPT1A induces phosphorylation and activation of the FoxO transcription factors Ovarian cancer cell lines were treated with etomoxir (0.3 mM) for the indicated periods of time (hours). Immunoblotting analyses were performed to assess phosphorylation of AMPKa and FoxO3a S413 (AMPK specific phosphorylation site) without or with Compound C (20 μM, 12 hours) (**A**), or Erk, p38, JNK and FoxO4 T451 (JNK specific phosphorylation site) (**B**). (**C**) DNA-binding activity of FoxO transcription factors in etomoxir-treated cells was assessed by EMSA. The FoxO consensus oligonucleotide was the native DNA sequence from the human p21 gene promoter (see Supplementary Table S1). The specificity of binding to the FoxO probe was confirmed by inhibition of binding with unlabeled oligonucleotide (cold competition) and loss of such binding to its mutant form.

AMPK activates FoxO proteins through direct phosphorylation of a number of serine and threonine residues [33, 35]. As shown earlier in Figure 2C, inactivation of CPT1A decreased cellular ATP levels and activated AMPK. Using an antibody for FoxO3a phosphorylated by AMPKα at S413 [35], we confirmed that etomoxir treatment increased FoxO3a phosphorylation at S413 in a pattern matching well with the kinetics of AMPKα phosphorylation in these cells (Figure 6A). The AMPK inhibitor compound C prevented etomoxir-induced FoxO3a S413 phosphorylation (Figure 6A).

Phosphorylation of FoxO by JNK and p38 kinases could also lead to activation of FoxO proteins [36–38]. Etomoxir treatment induced activation of p38 and JNK, but not Erk, as indicated by increased phosphorylation of p38 at T180/Y182 and JNK at T183/Y185 (Figure 6B). These kinases could lie downstream of AMPK that is known phosphorylate and activate p38 and JNK in certain conditions [39, 40]. However, p38 and JNK could be also activated by other mechanisms in FAO-depleted cells independently of AMPK. Indeed, their activation kinetics was not consistent with AMPK being a sole mediator of p38 or JNK activation in etomoxir-treated cells (Figure 6A, 6B). In Caov-3, phosphorylation of p38 was consistently elevated from 1.5 hours through 24 hours. Phosphorylation of p38 in SKOV-3 and OVCA-432 showed a biphasic pattern where a strong, early signal was detected at 1.5–3 hours and a second peak seen at 24 hours (Figure 6B). However, no phospho-specific antibodies against FoxO proteins phosphorylated by p38 are available for analysis of the potential input of p38 in FoxO activation.

Etomoxir-induced JNK phosphorylation occurred at 1.5 hours and declined after 3 hours in SKOV-3 and OVCA-432 or after 6 hours in Caov-3 (Figure 6B). T451 of FoxO4 is known to be specifically phosphorylated by JNK [36, 41]. Unlike commonly expressed FoxO1 and FoxO3a, FoxO4 is not present in SKOV-3 (Figure 6B). However, in FoxO4-expressing Caov-3 and OVCA-432 cells, etomoxir induced FoxO4 phosphorylation at T451 in a time-dependent manner consistent with JNK phosphorylating and activating FoxO proteins in these cells (Figure 6B).

The underlying mechanism for activation of the FoxO transcriptional activity is not fully understood. It is likely that multi-site phosphorylation promotes their nuclear translocation and DNA-binding activity [33, 41]. To test this, we performed EMSA to determine whether inhibition of CPT1A enhances DNA-binding activity of nuclear FoxO proteins. A 22-bp DNA sequence (CCAAAGTAAACAGACAGACAAT) from the native p21 gene promoter [27] was used as a probe. Nuclear extracts from etomoxir-treated cells were incubated with the ^32^P-labeled FoxO probe. Treatment with etomoxir stimulated time-dependent activation of binding of nuclear proteins to the probe but not to a mutant version lacking the FoxO consensus sequence (Figure 6C). In SKOV-3 and Caov-3, the binding of nuclear proteins to the probe was detected at 3 hours. The binding remained to be elevated by 24 hours in Caov-3 while the activity in SKOV-3 peaked at 12 hours. In OVCA-432, an earlier response was observed at 1.5 hours but diminished by 3 hours and a second-phase activity occurred at 6 hours and plateaued from 12 to 24 hours.

## DISCUSSION

Different from the well-defined pro-oncogenic role of lipogenesis, the biological relevance of lipid catabolism via FAO to cancer is not well understood. While considerable studies based on metabolomics analysis or pharmacological inhibitors have suggested involvement of FAO in cancer cells [5, 9, 42, 43], there is no direct evidence or molecular mechanism supporting the concept that cancer cells indeed rely on FAO for maintaining malignancy. In the present work, we investigated the biological relevance of FAO to ovarian cancer. Our results indicated that CPT1A, the rate-setting enzyme of FAO is abundantly expressed in a high percentage of ovarian serous carcinomas and ovarian cancer cell lines. Based on the analysis of pre-existing expression and patient outcome data, we found that CPT1A overexpression contributed to poor survival of ovarian cancer. Inactivation of CPT1A induced expression of the cyclin-dependent kinase inhibitor p21 and cell cycle arrest at G0/G1. Knockdown of CPT1A expression also suppressed tumorigenicity and aggressiveness of ovarian cancer cells in SCID mice. Mechanistic studies identified FoxO as a key transcription factor responsible for upregulation of p21. As further mechanism upstream of FoxO activation, inhibition of CPT1A stimulated phosphorylation of FoxO through activation of AMPK, p38 and JNK kinases. These results not only showed cellular consequences of blocking FAO but also elucidated a molecular mechanism coupling fatty acid catabolism to cell cycle progression and cancer pathogenicity, suggesting that the FAO pathway could be a viable target for therapeutic intervention in ovarian cancer and probably other human malignancies.

Previous studies have shown diverse cellular effects of etomoxir in different types of cancer cells [20–22, 44, 45]. In the HepG2 hepatocellular carcinoma cells, etomoxir at 1 mM was found to trigger both growth inhibition and apoptotic/necrotic cell death [44]. Schlaepfer et al. reported that treatment with etomoxir reduced viability of prostate cancer cells. The effects seemed to be associated with decreases in androgen receptor expression and mTOR signaling, as well as an increase in caspase-3 activity [22]. Another study in multiple myeloma cells by Tirado-Vélez et al. demonstrated that etomoxir inhibited proliferation via G0/G1 arrest [45], similar to the primary cellular effect we observed here in ovarian cancer cells. However, p21 was found to be downregulated by etomoxir in myeloma cells, suggesting that other cell cycle regulators might have mediated the anti-proliferative effect of etomoxir in myeloma cells [45]. In our study, etomoxir treatment and shRNA knockdown elicited strikingly similar effects on p21 and other cell cycle-related genes. Upregulation of p21 seems to be a major effector of CPT1A depletion although we can't rule out the possibility for involvement of additional unrecognized players in cell cycle arrest of ovarian cancer cells. Most recently, Schoors et al. reported that fatty acid carbon source from CPT1A-dependent FAO was essential for dNTP synthesis in endothelial cells [46]. Loss of CPT1A led to impaired proliferation of endothelial cells and vascular sprouting defects. This dNTP-mediated function of CPT1A seemed to be specific to endothelial cells, not shared by other cell types including epithelial cells [46]. In contrast to our observation of ATP downregulation and AMPK activation in ovarian cancer cell lines, these authors did not observe ATP depletion in CPT1A-inactivated endothelial cells [46], suggesting complex roles of CPT1A and FAO in different cell types.

In physiological conditions, lipogenesis and FAO are mutually exclusive processes, controlled by the level of malonyl-CoA. Malonyl-CoA is an intermediate of fatty acid synthesis and meanwhile an allosteric inhibitor of CPT1, preventing fatty acid biosynthesis and FAO from occurring simultaneously. However, this regulation seems to be defective in cancer cells if we and others are right in that both lipid anabolism and catabolism are highly active in cancer. The mechanism subverting the tight control of lipid anabolic and catabolic processes by malonyl-CoA in cancer cells is not fully understood. One possibility is that CPT1C, a subtype of CPT1 lacking enzymatic activity but capable of binding and quenching malonyl-CoA, might be highly expressed, preserving the enzymatically active CPT1A in malignant cells [13, 14]. In the present study, however, we observed abundant expression of CPT1A, but not other CPT1A isoforms in ovarian cancer cells. Increased CPT1A levels could provide a direct means to counteracting the inhibitory effect of malonyl-CoA on activity of CPT1A, allowing active lipogenesis and FAO to take place concurrently.

We further asked how inhibition of CPT1A culminated in p21 induction. Our experiments provide multilevel evidence pointing to FoxO as an effector downstream of CPT1A inhibition. The FoxO transcription factors are important mediators of not only tumor suppression but also longevity as demonstrated in C. elegans [47, 48]. FoxO regulates expression of a broad range of targets including microRNAs [49]. In addition to phosphorylation-dependent regulation of FoxO, the stability and activity of FoxO could be also modulated by methylation [50]. Our study demonstrated that the FoxO transcription factors were required for upregulation of p21 transcription in CPT1A-depleted cells, as suggested by mutational analysis of the p21 gene promoter as well as shRNA silencing of FoxO. Furthermore, we observed that several intracellular kinases including AMPK, p38 and JNK became phosphorylated/activated in response to inhibition of CPT1A. These are kinases known to phosphorylate FoxO proteins at specific sites, leading to activation of their DNA-binding and/or transcriptional activities [33, 36]. Our data indicate that these kinases participate in phosphorylating FoxO transcription factors to enhance FoxO transcriptional activity and p21 expression in CPT1A-depleted cells.

In sum, CPT1A is abundantly expressed in ovarian cancer. Its overexpression contributes to poor prognosis of the disease. Ovarian cancer cells rely on or are “addicted” to CPT1A activity for maintaining growth and the malignant phenotype. The CPT1A-dependent FAO functions to restrain the FoxO transcriptional activity in ovarian cancer cells. Disruption of such a control leads to phosphorylation and activation of FoxO by AMPK, JNK and p38, and the subsequent induction of p21 and cell cycle arrest at G0/G1. These results reveal oncogenic relevance of CPT1A and a mechanistic link from lipid catabolism to cell cycle regulation in ovarian cancer.

## MATERIALS AND METHODS

### Reagents

Fetal bovine serum (FBS) was obtained from Atlanta Biological (Atlanta, GA). Epidermal growth factor (EGF), gentamicin and etomoxir were purchased from Sigma-Aldrich (St. Louis, MO). Antibodies used for western blotting against CPT1A, p21, p-FoxO3a (S413), p-FoxO4 (T451), p-p38 (T180/Y182), p-JNK (T183/Y185), p-Erk1/2 (T202/Y204), p-AMPK (T172), and β-actin were obtained from Cell Signaling (Beverly, MA). A CPT1A mouse monoclonal antibody and Ki67 rabbit monoclonal antibody for IHC and compound C were purchased from Abcam (Cambridge, MA).

### Cells

The culture conditions for ovarian cancer cell lines and normal ovarian epithelial (NOE) cells were described previously [51–53]. These cells were either from ATCC or from Dr. GB Mills and Dr. RC Bast, Jr. (MD Anderson Cancer Center). They were frozen at early passages and used for less than a month in continuous culture.

### Ovarian cancer specimens and IHC staining

Ovarian cancer patients were diagnosed at the Virginia Commonwealth University (VCU) Hospitals. Tumor tissues were collected during surgeries for diagnostic and therapeutic purposes. These preexisting specimens were not obtained specifically for the present study. With appropriate IRB approvals, paraffin-embedded sections of 18 randomly selected high-grade ovarian serous carcinomas (stage II–IV) were assessed by IHC for expression of CPT1A using an IHC kit (VECTASTAIN Elite ABC Kit, Vector Laboratories, Burlingame, CA) according to the manufacturer. The sections were examined by a pathologist (M Idowu) and two more persons in the group, and classified based on percentages of positive cells: 0, < 10%; 1, 10–25%; 2, 25–50%; 3, 50–75%; and 4, 75–100% and staining intensity (0, negative; 1, weak; 2, moderate; and 3, strong) [54]. The staining was scored by multiplying the percentage classification by the intensity.

### Quantification of cellular ATP

After trypsinization and washing twice with PBS, cells were resuspended at 1 × 10^6^ cells/ml of H_2_O containing 0.75% NP-40 and vertexed at full speed for 10 seconds, and incubated on ice for 10 minutes. After centrifugation (16,000 g, 3 minutes), the supernatants were collected for ATP measurement with an ATP bioluminescence assay kit (Sigma-Aldrich). ATP concentrations were calculated from the standard curve and presented as nMol of ATP per 10^6^ cells.

### Plasmids and luciferase assay

The pGreenPuro shRNA plasmid and control shRNA were described previously [55]. The shRNA lentivirus vectors were generated by cloning target-specific oligonucleotides into the pGreenPuro shRNA plasmid. The target sequences of shRNA template oligonucleotide used were listed in Supplementary Table S1. The p21 gene promoter in WWP-Luc was obtained from Dr. B Vogelstein [27] and re-cloned into pGL2-Basic-Luc (Promega, Madison, WI) to make pGL2-p21-Luc. The FoxO site in pGL2-p21-Luc was mutated to generate pGL2-p21mu-Luc by overlapping PCR with primers listed in Supplementary Table S1.

Cells seeded in 6-well plates were transfected with luciferase plasmids using transIT-IL1 transfection reagent (Mirus Bio Corp., Madison, WI) according to the manufacturer. After 48 hours, the cells were treated with etomoxir or vehicle (water) for 24 hours. The cells were then lysed and assayed for luciferase activity using a kit from Promega.

### Western blotting

Cells were lysed in SDS sample buffer or in ice-cold cell lysis buffer (Cell Signaling Technology). Cellular proteins were separated on SDS-PAGE and transferred to polyvinylidene difluoride membrane (Bio-Rad, Hercules, CA) and immunoblotted with antibodies following the protocols of manufacturers. Immunocomplexes were visualized with an enhanced chemiluminescence detection kit.

### Lentivirus preparation

The packaging of VSV-glycoprotein pseudotyped lentiviral particles were described previously [55]. Viruses were produced by co-transfection of 293TN cells with a lentiviral vector, packaging and envelope plasmids using lipofectamine 2000 (Life Technology). The virus-containing supernatants were harvested 2–3 days post transfection and used for infection of cells or stored in aliquots at −80°C.

### Electrophoretic mobility shift assay (EMSA)

Etomoxir-treated or control cells were harvested and nuclear proteins were extracted as we described previously [52]. Protein concentrations were determined with the Pierce BCA protein assay kit before EMSA.

The FoxO consensus oligo and its mutant form (Supplementary Table S1) were labeled at 3′-ends with [α-^32^P] dCTP with Klenow. Nuclear proteins (4 μg) were briefly incubated in 25 ul of binding buffer [10 mM HEPES (pH 7.8), 1 mM EDTA, 5 mM MgCl2, 50 mM KCl, 10% glycerol, 3 μg poly (dI-dC), and 4 μg BSA]. The labeled oligonucleotides or cold competitor (with 50-fold excess of unlabeled oligonucleotides) was added to the reaction and incubated for an additional 15 min at room temperature before electrophoresis on 5% native polyacrylamide gels. The gels were dried and subjected to autoradiography using a Phosphorimager.

### mRNA analysis

Total cellular RNA was extracted from cells in culture with TRIzol reagent (Life Technologies, Carlsbad, CA). Complementary DNA (cDNA) was synthesized by using the SuperScript III First-Strand Synthesis kit (Life Technologies). The relative levels of CPT1A mRNA were determined by qPCR using CPT1A-specific probe, the TaqMan Universal PCR master mix, and the Applied Biosystems 7900HT real time PCR. Glyceraldehyde-3-phosphate dehydrogenase (GAPDH) was used as an internal control for normalization.

For the Cell Cycle PrimePCR Assay (BioRad), total cellular RNA was treated with DNase before synthesis of cDNA with the SuperScript III First-Strand Synthesis system. The relative gene expression levels were determined by qPCR with SYBR^®^ Green and results were normalized to that of GAPDH and then compared with the control cells.

### [^3^H] Thymidine incorporation assay

Cellular DNA synthesis was measured by [^3^H] thymidine incorporation. Cells in 12-well plates were cultured in fresh medium with or without etomoxir for 24 hours and pulse-labeled with [^3^H] thymidine (PerkinElmer, Boston, MA) (1 μCi/well) for the last 3 hours. At the end of labeling, the cells were washed twice with PBS, twice with 5% cold trichloroacetic acid (30 and 5 minutes at 4°C), and once with 95% ethanol. Trichloroacetic-acid-insoluble material was dissolved in 0.2 M sodium hydroxide overnight and then scintillation-counted for radioactivity.

### Cell cycle analysis

At approximately 50–60% confluence, CPT1A knockdown cells, etomoxir-treated cells (0.3 mM, 24 hours) and control cells were trypsinized, washed twice with PBS, and resuspended at a concentration of 1 × 10^6^ cells/ml in a fluorochrome staining solution [56] and incubated on ice for 3 hours or kept at 4°C for up to 5 days prior to flow cytometric analysis using BD FACSCanto II Analyzer (BD Bioscienses, San Jose, CA).

### Senescence-associated β-galactosidase (SA-β-Gal) staining

The assay was performed as previously described [24]. The cells were washed in PBS, fixed in 4% formaldehyde for 5 min, and incubated for up to 16 hours with freshly made SA-β-Gal staining solution [1 mg/ml X-Gal, 40 mM of citric acid/sodium phosphate, pH 6.0, 5 mM potassium ferrocyanide, 5 mM potassium ferricyanide, 150 mM NaCl and 2 mM MgCl_2_].

### Soft agar assay

To assess the capability of CPT1A knockdown and control cells to form colonies in anchorage-independent conditions, cells were resuspended in 0.3% soft agar/complete medium and seeded at 5000 cells/1.5 ml in 6-well plate pre-coated with 1.5 ml of 0.6% soft agar/complete medium. Cells were cultured for 3 weeks to allow colony formation. Fresh medium was gently overlayered to the wells every 5 days.

### Xenograft models in SCID mice

CPT1A knockdown and control cells were cultured in 100-mm dishes. The cells in exponential growth phase were trypsinized, washed twice with PBS and resuspended in the serum-free medium. The cells (1 × 10^6^ of SKOV-3 and 2 × 10^6^ of OVCAR-3 or OVCA-432) were injected subcutaneously (s.c.) on the right flank of NOD CB17 SCID/SCID mice (female, 6–7 weeks old). The formation of subcutaneous tumors was monitored and measured with a digital caliper. The tumor volumes were calculated based on the formula *lw*^2^/2 where *l* is the length and *w* is the shortest width of the tumor. CPT1A-sh2 knockdown SKOV-3 cells and ctrl-sh control cells were also i.p. injected into SCID mice (10×10^6^/mouse, *n =* 6). The development of tumors and ascites and survival of mice were monitored on daily basis from 8 days after cell injection as we described previously [57]. All animal experiments were conducted in compliance with the policies and regulations of VCU IACUC.

### Statistics

All numerical data were presented as mean ± SD. The statistical significances of differences were analyzed using Student's *t*-test except that Kaplan Meier survivals of human patients and mice were analyzed with Gehan-Breslow-Wilcoxon test and LogRank test, respectively. In all tests, differences with *p* values < 0.05 were considered to be statistically significant.

## SUPPLEMENTARY MARTERIAL FIGURES AND TABLE



## Abbreviations

CPT1: carnitine palmitoyltransferase 1
FAO: fatty acid β-oxidation
FAS: fatty acid synthase
ACC: acetyl-CoA carboxylase
FoxO: forkhead box O
GAPDH: glyceraldehyde-3-phosphate dehydrogenase
p21: p21^WAF1^
AMPK: AMP-activated protein kinase
NOE: normal ovarian epithelial
IHC: immunohistochemical or immunohistochemistry
β-gal: β-galactosidase
SA-β-Gal: Senescence-associated β-galactosidase
EMSA: electrophoretic mobility shift assay.
