# Supplementary material for: Carnitine palmitoyltransferase 1A functions to repress FoxO transcription factors to allow cell cycle progression in ovarian cancer
Source: Oncotarget. 2015 Dec 24;7(4):3832–46. doi: 10.18632/oncotarget.6757 (PMC4826173; doi:10.18632/oncotarget.6757)
Supplement: Supplementary file 1 [file oncotarget-07-3832-s001.pdf]

Carnitine palmitoyltransferase 1A functions to repress FoxO transcription factors to allow cell cycle progression in ovarian cancer

Supplementary Materials

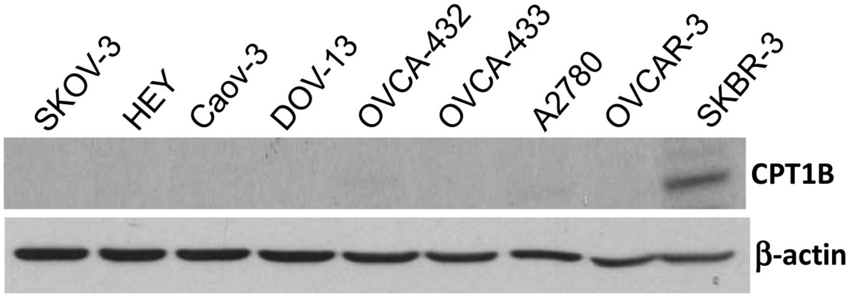

Supplementary Figure S1: Lack of CPT1B in ovarian cancer cell lines. Expression of CPT1B protein in ovarian cancer cell lines was examined by Immunoblotting with a polyclonal antibody from Abcam (ab134988). The CPT1B- expressing SKBR-3 breast cancer cell line was included as a positive control.

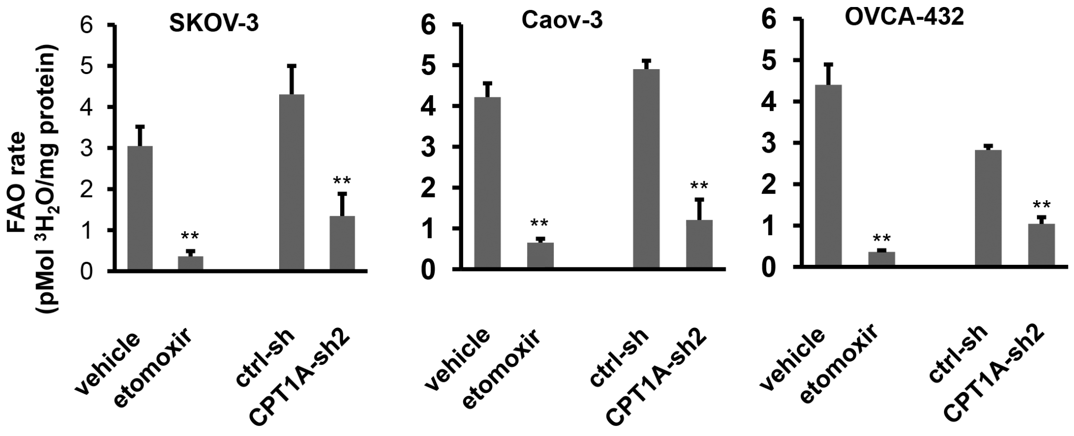

Supplementary Figure S2: Inactivation of CPT1A inhibits FAO. CPT1A was inactivated by CPT1A-sh2 knockdown or by treatment with etomoxir (0.3 mM, 18 hours). The cells in 12-well plates were incubated for 3 hours in 500 µL of the palmitic acid-BSA mixture in Kreb's buffer containing 22 µM sodium palmitate, 7.5 µM fatty acid-free BSA and 2.5 µCi [9,10–3H(N)] palmitic acid (PerkinElmer, Waltham, MA). The culture medium was collected and mixed with equal volume of 10% trichloroacetic acid. After centrifugation at 16,000g for 5 minutes, supernatants (500 µL) were mixed with 100 µL of 6N NaOH and then loaded to columns packed with AG 1X8, 100–200 mesh ion exchange resin (Bio-Rad, Hercules, CA). The radioactivity present in eluted water was measured with liquid scintillation counting. The results were presented as pMol of 3H2O per mg of cellular proteins.

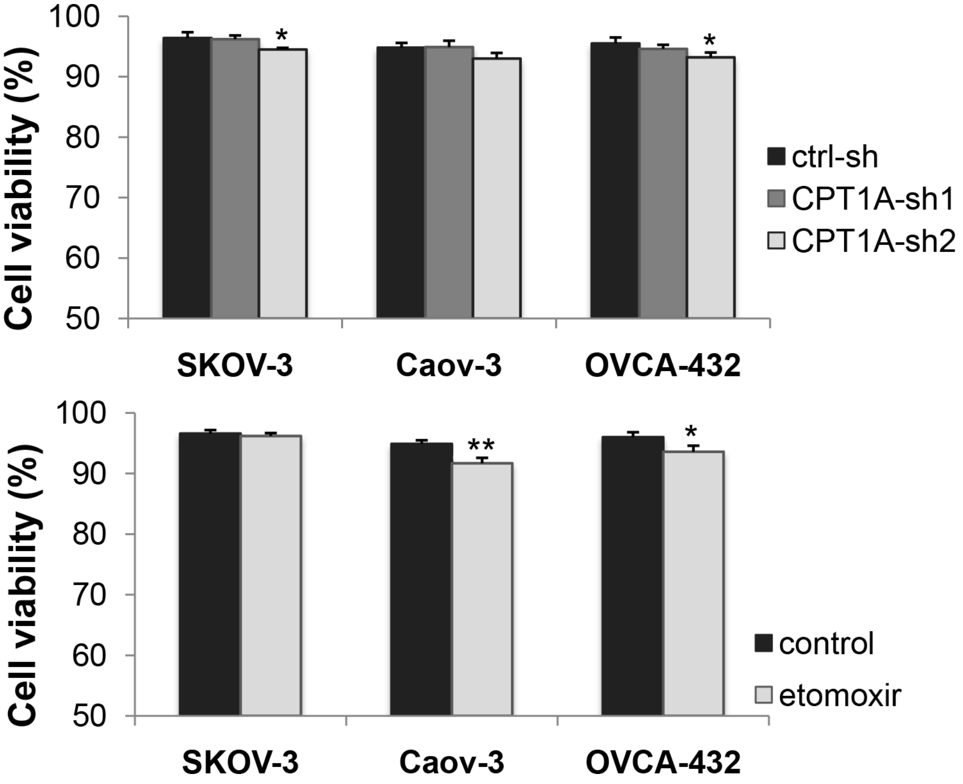

Supplementary Figure S3: The Effect of CPT1A inactivation on apoptosis was determined in CPT1A shRNA knockdown cells (upper) and in parental cell lines treated with etomoxir (0.3 mM for 24 hours) (lower). Both attached and floating cells were collected for staining of apoptotic cells with FITC-conjugated Annexin V (Fisher Scientific). The viabilities were determined with fluorescence microscope by counting Annexin V-positive populations from more than 250 cells of triplicate wells. There were little or slight decreases in cell viability (< 4%) associated with CPT1A inactivation.

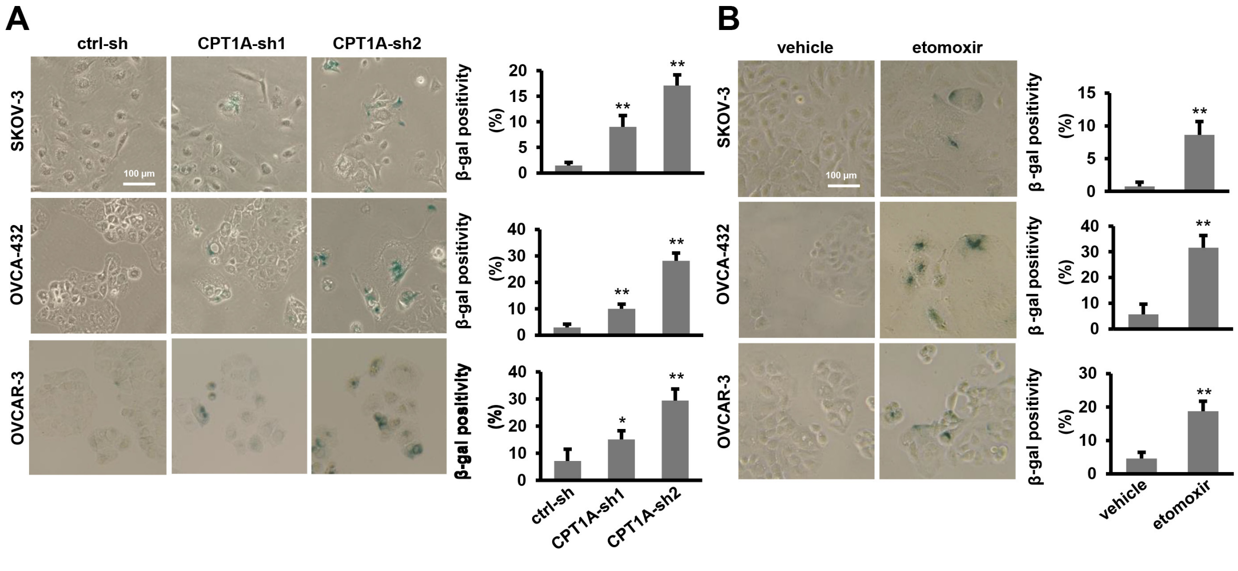

Supplementary Figure S4: CPT1A inactivation triggers replicative senescence in SKOV-3, OVCA-432 and OVCAR-3 cells. (**A**) After lentivirus infection and brief selection with puromycin (2 µg/ml) for 5 days, SKOV-3, OVCA-432 and OVCAR-3 cells were stained for SA-β-gal activity. After staining, representative fields of ctrl-sh, CPT1A-sh1, and CPT1A-sh2-transduced cells were photographed under microscope. Percentages of SA-β-gal-positive cells in triplicate dishes were quantified by Image J software. (**B**) SA-β-gal staining was conducted in the three cell lines after treatment for 3 days with or without etomoxir (0.3 mM).

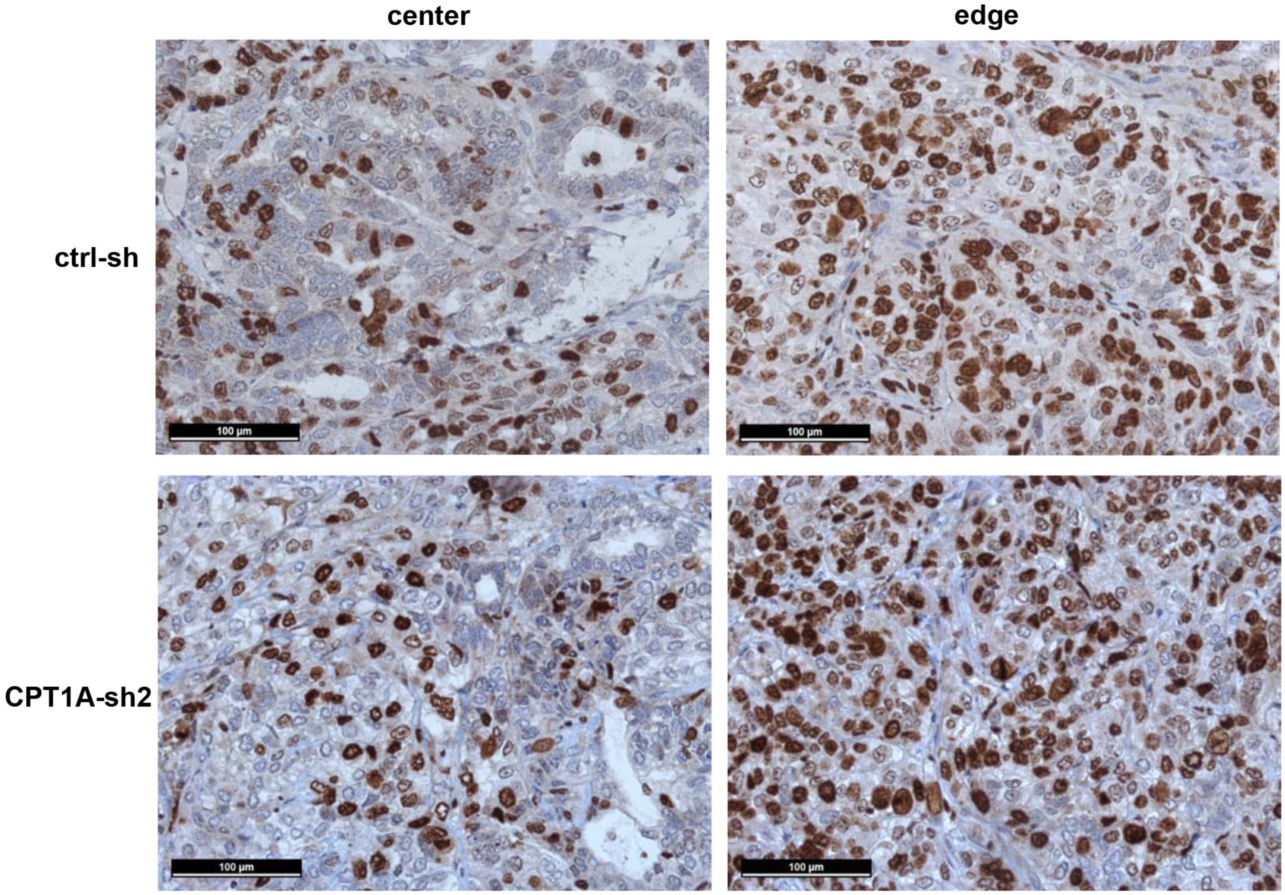

Supplementary Figure S5: IHC staining of Ki67 in xenografts. Shown were representative s.c. xenografts grown from CPT1A-sh2 knockdown SKOV-3 cells (lower panels) and ctrl-sh SKOV-3 cells (upper panels). More cells in peripheral regions (right panels) were stained positive for Ki67 than those in central regions (left panels) of xenografts.

Supplementary Table S1: Oligos used in the work

CPT1A-sh1	5′- GCCTCTTATGAAGGAAGAAGA -3′
CPT1A-sh2	5′- GGGAGTACGTCATGTCCATTG -3′
FoxO1-sh	5′- GAGCGTGCCCTACTTCAAG -3′
FoxO3a-sh	5′- GTCACTGCATAGTCGATTCAT -3′
FoxO WT probe	5′- GGCCAAAGTAAACAGACAGACAAT -3′ (forward)<br>5′- GGATTGTCTGTCTGTTTACTTTGG -3′ (reverse)
FoxO mut probe	5′- GGCCAAAGAATTCAGACAGACAAT -3′ (forward)<br>5′- GGATTGTCTGTCTGAATTCTTTGG -3′ (reverse)
